# Pre-labor silent rupture of unscarred uterus at 32 weeks with intact amniotic sac extrusion: a case report

**DOI:** 10.4076/1757-1626-2-7095

**Published:** 2009-07-16

**Authors:** Ritu Rana, Manju Puri

**Affiliations:** 1 Department of Obstetrics & Gynecology, Princess Alexandra HospitalHarlow, Essex, CM20 1QXUK; 2 Department of Obstetrics & Gynecology, Lady Hardinge Medical College and Smt Sucheta Kriplani HospitalNew Delhi 110 001India

## Abstract

**Introduction:**

Spontaneous rupture of uterus in unscarred uterus prior to onset of labor in third trimester is extremely rare and to our knowledge, very few cases have been reported so far.

**Case presentation:**

A 26-year-old third gravida women with 32 weeks pregnancy presented with pre-labor rupture uterus with extrusion of intact amniotic sac from the rent in uterine fundus.

**Conclusion:**

Rupture uterus can present in third trimester even before labor and should be kept in differential diagnosis of pregnancy with abdominal pain of any degree with fetal demise.

## Introduction

Spontaneous rupture of uterus has been reported many times in pregnant women mainly during labor, due to external injuries and in scarred uterus. However, spontaneous rupture in unscarred uterus prior to onset of labor in third trimester is extremely rare [1-3].

## Case presentation

A 26-year-old pregnant women of Asian Indian origin was referred to the obstetric casualty by the general practitioner with a provisional diagnosis of placental abruption in view of abdominal pain, pallor and inability to find fetal heart. The women had insidious onset of pain for 12 hrs, and with reduced fetal movements for the same duration. The woman was third gravida with 32 weeks gestation, with previous two normal deliveries and an uneventful antenatal period. Her dating and anomaly scan were normal. There was also no history of trauma, any diagnostic or therapeutic intrauterine intervention, vaginal bleeding or labor pains. She had no significant past medical, surgical history or gynecological history.

On examination, she was pale with respiratory rate of 28 per minute, pulse rate of 110 per minute and blood pressure of 90/60 mmHg. On abdominal examination, uterus measured 30 weeks, with mild abdominal tenderness and no increased tone. The uterine wall felt thin but with regular outline and no fetal part was palpable. Fetal heart could not be localized. Speculum examination showed no vaginal bleeding and there was marked tenderness on internal examination and cervix was uneffaced and os admitted tip of finger.

Ultrasound scan revealed fetal demise. An intact amniotic sac with fetus and normal volume of amniotic fluid, without any echogenecity to suggest blood, was seen lying outside the uterus. Uterus was deviated towards left iliac fossa. Diagnosis of rupture uterus with fetal demise was made and the woman was prepared for laparotomy. Blood investigations revealed hemoglobin of 7.7 gm% with no evidence of coagulopathy.

After resuscitation with intravenous fluids, the patient was taken for surgery. Emergency exploratory laparotomy was done with consent for hysterectomy or uterine repair with tubal sterilization under intravenous antibiotic cover. An intact amniotic sac with fetus was found along with 1.5 liter of haemoperitoneum. Membranes were ruptured and male stillborn fetus (weight 2.2 kilograms) was delivered with breech extraction. The amniotic fluid was clear, and placenta was lying outside the uterus and appeared complete and normal. There was a 3 inch tear on the right side of the uterine fundus, 2 cm anterior to the cornua. The myometrium adjacent to this was relatively thin. There was no evidence of couvelier uterus. The tear was repaired by suturing the uterus in double layer. Bilateral tubal ligation was done. She received one unit of blood during intra-operative and three during the postoperative period. Her postoperative period was uneventful and was discharged after eight days.

## Discussion

Rupture of unscarred uterus is a rare event involving 1:16,000 deliveries [4]. The probable causes in reported cases are external injuries, induction of labor, high birth order, cephalo-pelvic disproportion, placenta accreta, fundal pressure, abruption, cocaine abuse and history of intrauterine intervention causing perforation [5-9].

To our knowledge, this is the fourth reported case of pre-labor silent rupture uterus in third trimester in an unscarred uterus. In one of the reported cases, rupture uterus was found behind the cornual structures and was diagnosed on laparotomy done for deteriorating maternal condition after vaginal delivery of the stillborn baby by forceps [1]. The second reported case had a tear above the insertion of right uterosacral ligament and the baby was born by emergency lower segment Caesarean section [2]. The third case reported posterior uterine wall tear diagnosed on laparotomy done for post failed induction for deteriorating maternal condition and fetal demise [3].

Apart from pre-labor uterine rupture in an unscarred uterus, this case had a subacute onset. The fetus along with the amniotic sac had silently extruded through the rent in uterus making the diagnosis of rupture uterus difficult clinically, as uterine contour was falsely preserved due to intact amniotic sac. There was no placenta accreta, couvelaire uterus, any signs of placental abruption or history of connective tissue disorder like Ehler Danlos syndrome [9]. It thus makes us speculate that this rupture could have been due to some inherent weakening in the uterine myometrium, which became more profound in third pregnancy due to repeated stretching from previous pregnancies.

## Conclusion

This case re-emphasizes that rupture uterus can present in many different ways and high suspicion is required for timely intervention to prevent maternal morbidity and mortality.

## References

[bib-001] Abbi M, Misra R (1997). Rupture of uterus in primigravida prior to onset of labor. Int J Fertil Womens Med.

[bib-002] Langton J, Fishwick K, Kumar B, Nwosu EC (1997). Spontaneous rupture of an unscarred gravid uterus at 32 weeks gestation. Human Rep.

[bib-003] Walsh CAMB, O’Sullivan RJ, Foley ME (2006). Unexplained prelabor uterine rupture in a term primigravida. Obstet Gynecol.

[bib-004] Miller DA, Paul RH (1996). Rupture of the unscarred uterus. Am J Obstet Gynecol.

[bib-005] Turner MJ (2002). Uterine rupture. Best Pract Res Clin Obstet Gynaecol.

[bib-006] Imseisss HM, Murtha AP, Alexander KA, Barnett BD (1998). Spontaneous rupture of primigravidae uterus secondary to Placenta Percreta. J Reprod Med.

[bib-007] Schrinsky DC, Benson RC (1978). Rupture of pregnant uterus: a review. Obstet Gynecol Surv.

[bib-008] Gonsoulin W, Borge D, Moise KJ (1990). Rupture of unscarred uterus in primigravid woman in association with cocaine abuse. Am J Obstet Gynecol.

[bib-009] Gelbmann CM, Kollinger M, Gmeinwieser J, Leser HG, Holstege A, Scholmerich J (1997). Spontaneous rupture of liver in a patient with Ehlers Danlos disease type IV. Dig Dis Sci.

